# Positive autoregulation of the transcription factor Pax6 in response to increased levels of either of its major isoforms, Pax6 or Pax6(5a), in cultured cells

**DOI:** 10.1186/1471-213X-6-25

**Published:** 2006-05-25

**Authors:** Jeni Pinson, T Ian Simpson, John O Mason, David J Price

**Affiliations:** 1 Genes and Development Group, Centre for Integrative Physiology, University of Edinburgh, Hugh Robson Building, George Square, Edinburgh EH8 9XD, UK

## Abstract

**Background:**

Pax6 is a transcription factor essential for normal development of the eyes and nervous system. It has two major isoforms, Pax6 and Pax6(5a), and the ratios between their expression levels vary within narrow limits. We tested the effects of overexpressing either one or other isoform on endogenous Pax6 expression levels in Neuro2A and NIH3T3 cells.

**Results:**

We found that both isoforms caused an up-regulation of endogenous Pax6 expression in cells with (Neuro2A) or without (NIH3T3) constitutive Pax6 expression. Western blots showed that cells stably transfected with constructs expressing either Pax6 or Pax6(5a) contained raised levels of both Pax6 and Pax6(5a). Quantitative RT-PCR confirmed an increase in levels of *Pax6(5a) *mRNA in cells containing Pax6-expressing constructs and an increase in levels of *Pax6 *mRNA in cells containing Pax6(5a)-expressing constructs. The fact that the introduction of constructs expressing only one isoform increased the cellular levels of not only that isoform but also the other indicates that activation of the endogenous *Pax6 *locus occurred. The ratio between the levels of the two isoforms was maintained close to physiological values. The overexpression of either isoform in neuroblastoma (Neuro2A) cell lines also promoted morphological change and an increase in β-III-tubulin expression, indicating an increase in neurogenesis.

**Conclusion:**

Our results demonstrate that Pax6 can up-regulate production of Pax6 protein from an entire intact endogenous *Pax6 *locus in its genomic environment. This adds to previous studies showing that Pax6 can up-regulate reporter expression driven by isolated *Pax6 *regulatory elements. Furthermore, our results suggest that an important function of positive feedback might be to stabilise the relative levels of Pax6 and Pax6(5a).

## Background

The Pax6 transcription factor contains a paired domain and a homeodomain and is expressed in a complex spatio-temporal pattern during development of the retina, lens and cornea, in regions of the forebrain, hindbrain, cerebellum and spinal cord, the olfactory system and in pancreatic islet cells [1-6]. Haploinsufficiency for *Pax6 *function (*Pax6*^+/-^) in the mouse results in the *Small eye *(*Sey*) phenotype. Homozygotes (*Pax6*^-/-^) die perinatally with no eyes, no nasal structures and many severe brain abnormalities [4,6-17].*PAX6 *haploinsufficiency also causes eye and brain defects in humans [18,19]

Pax6(5a) was the first isoform of the *Pax6 *gene to be described [5] and is generated by the alternative splicing of exon 5a. The inclusion of this exon disrupts the paired domain and alters its DNA binding properties, potentially allowing Pax6 to activate a different set of downstream target genes [20,21] Although the function of the Pax6(5a) isoform has not yet been determined, a number of studies have attempted to address the phenotypic implications of this alternative splicing event. For example, in the eye, constitutive deletion of murine exon 5a leads to iris hypoplasia, alongside defects in the development of the cornea, lens and retina [22]. Conversely, overexpression of Pax6(5a) in the mouse lens leads to the formation of cataracts and up-regulation of a number of cellular adhesion molecules [23], while Pax6(5a) overexpression in the chick retina induces hyperplasia of the neural retina [24]. Evidence from studies in vertebrates suggests that the functions of Pax6 and Pax6(5a) differ [25-27], which mirrors differences between the functions of their homologues in invertebrates [28,29].

Two promoters are thought to be responsible for the majority of *Pax6 *expression. They are known as P0 and P1 in quail and mouse [30-33](.(and are homologous to PA and PB in the human [34]. A series of experiments have indicated that when these elements are isolated and cloned into reporter plasmids they can be bound by Pax6 proteins themselves. Constructs expressing quail Pax6 homologues increase activity from constructs containing reporter genes driven either by the P0 or by the P1 promoter when these are introduced into quail neuroretina cells [32,33] Similarly, human PAX6 proteins affect the activity of isolated PB promoter, although the nature of the effect varies between cell lines [34], and murine Pax6 proteins activate transcription of a reporter gene *via *P0 [35]. These studies suggest that Pax6 proteins might positively autoregulate the gene. A crucial step is to discover the nature of Pax6 autoregulation in an intact locus rather than isolated elements since, on this point, evidence is currently very limited. Several studies have shown that Pax6 is required in some regions of the embryo (the lens, olfactory placodes and diencephalon) for *Pax6 *transcription [1,35,36], but whether the introduction of Pax6 it is sufficient to induce or upregulate expression from the intact locus is not known.

To search for evidence of positive autoregulation of an intact locus, we generated murine neuroblastoma and fibroblast cell lines [37,38]. with stable integration of constructs generating either one or other of the major isoforms of Pax6 [Pax6 or Pax6(5a)].

## Results

**Figure 1:**
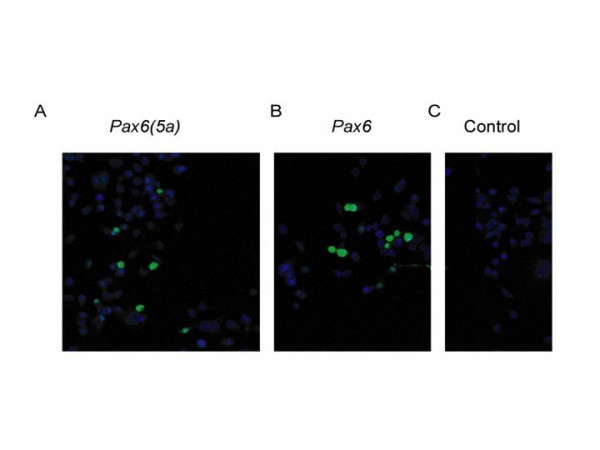
**Transfection of Neuro2A cells with Pax6(5a) and Pax6 expression constructs**. (A,B) Immunocytochemical detection of Pax6 proteins (green) following transient transfection of Neuro2A cells with *Pax6(5a) *or *Pax6*. (C) Untransfected Neuro2A cells do not express detectable levels of Pax6.

**Figure 2:**
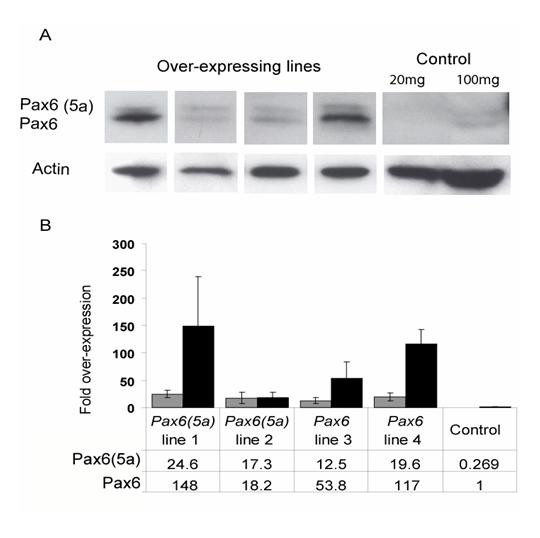
**Quantification of Pax6 and Pax6(5a) in over-expressing cell lines**. (A) Western blots showing Pax6(5a) and Pax6 production in four over-expressing stably transfected lines (20 mg protein per lane). In control lines, Pax6 proteins were not detectable unless 100 mg of protein were loaded per lane. (B) Quantification by densitometry of over-expression of Pax6(5a) and Pax6 in four over-expressing cell lines: lines 1 and 2 contained *Pax6(5a) *constructs, lines 3 and 4 contained *Pax6 *constructs. All values were expressed relative to levels of Pax6 in a control cell line. Mean values ± SEMs are plotted for levels of Pax6(5a) (grey bars) and Pax6 (black bars); all values, given below the histograms, are significantly higher than in controls (unpaired Student's *t*-tests: p < 0.01).

Neuro2A cells express *Pax6 *at low levels. Pax6 proteins were not detected reliably with immunocytochemistry in untransfected cells (Fig. 1C) but transfection with constructs expressing Pax6 or Pax6(5a) produced cells that were strongly immunoreactive (examples of transiently transfected cells are shown in Fig. 1A,B). Following selection, cell lines with stable integration of constructs expressing either Pax6(5a) or Pax6 were generated. Western blots showed that these over-expressing lines produced increased levels of Pax6 proteins (Fig. 2A). Pax6 proteins were only detectable in Western blots on control Neuro2A lines when the amount of protein loaded per lane was increased 5-fold (right-hand lane in Fig. 2A).

**Figure 3:**
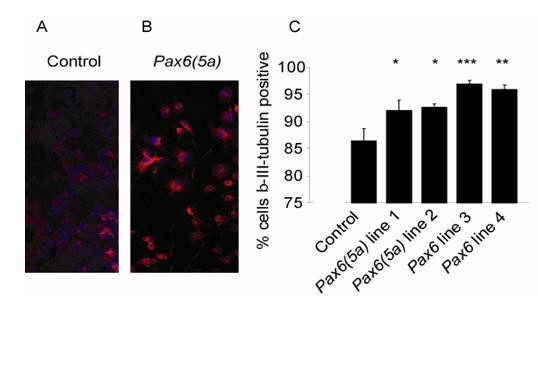
**Cell lines over-expressing Pax6(5a) or Pax6 show increased expression of a neuronal marker**. (A,B) β-III-tubulin expression in a control cells line and in a *Pax6(5a) *over-expressing cell line. (C) Quantification of β-III-tubulin expression in a control cell line and in four over-expressing lines: mean percentages of expressing cells (± SEMs) with results of unpaired Student's *t*-tests against untransfected cells indicated above bars (* = p < 0.05, ** = p < 0.01, *** = p < 0.001).

The ability of Pax6 over-expressing Neuro2A cells to differentiate along a neuronal pathway was assessed, to ascertain whether the responses of Neuro2A cells to an increased Pax6 level mimicked known responses of primary cells to Pax6 over-expression. *Pax6 *regulates neuronal differentiation and its over-expression in brain cells promotes neurogenesis [39]. Neuro2A cells can spontaneously differentiate along a neuronal pathway in culture [40] and we tested whether over-expression of Pax6 proteins would promote this process. Following seeding onto glass coverslips or plastic wells, the morphology of over-expressing cells was altered from that of control cells, in that they appeared larger and had longer neurites, and their levels of expression of the neuronal marker β-III-tubulin were higher (Fig. 3A,B). The percentage of cells expressing the neuronal marker, β-III-tubulin, was higher in all four over-expressing lines [two carrying *Pax6 *and two carrying *Pax6(5a) *constructs] than in control lines expressing green fluorescent protein (GFP) alone (i.e. transfected with pEGFPN1) [Fig. 3C]. We conclude that over-expression of Pax6 proteins promotes neurogenesis in Neuro2A cells, as it does in primary brain cells [39].

We then quantified the levels of Pax6 expression in four over-expressing Neuro2A cell lines and in a control Neuro2A cell line using densitometry on Western blots of a series of 3–4 protein extracts from each line (Fig. 2B). The density of each Pax6 and Pax6(5a) band was reported relative to that of the β-actin band for that lane and the fold over-expression was calculated relative to a value of 1 for the Pax6 band in the control line. We found that stable introduction of constructs expressing either *Pax6 *or *Pax6(5a) *increased production of not only the corresponding form of the protein, but also the other form (Fig. 2B). The increased production of both forms following introduction of constructs that could make only one form indicates that endogenous production must have increased. The introduction of Pax6 expression constructs (lines 3 and 4 in Fig. 2B) increased production of Pax6(5a) but levels remained below those of Pax6. Introduction of Pax6(5a) expression constructs (lines 1 and 2 in Fig. 2B) increased production of Pax6 to levels above those of Pax6(5a) in one line and equal to those of Pax6(5a) in the other. None of the cell lines expressed more Pax6(5a) than Pax6. The Pax6:Pax6(5a) ratios in the lines varied from 1.1:1 (line 2, Fig. 2B) to 6.0:1 (lines 1 and 4, Fig. 2B). We concluded that endogenous production can respond positively to an increased level of either Pax6 or Pax6(5a) protein and that the end-result is to maintain Pax6:Pax6(5a) ratios close to those seen in brain cells *in vivo*, which range from around 3:1 to around 10:1 depending on age and tissue type [41].

**Figure 4:**
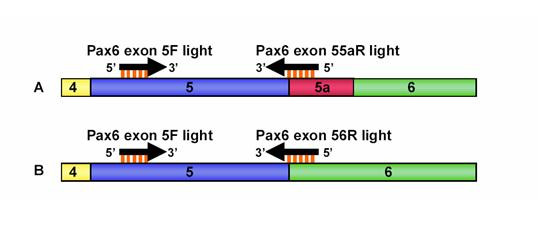
**Q-PCR primers used to distinguish *Pax6(5a) *from *Pax6***. One forward primer, "Pax6 exon 5F light", is used for both reactions, as it binds to sequence within exon 5 of the Pax6 cDNA. (A) The reverse primer "Pax6 exon 55aR light" anneals to sequence spanning the junction between exons 5 and 5a. The 3' 3 bp of the primer, which are crucial for primer binding, cross the exon boundary. If exon 5a is absent, this primer can not bind. (B) The reverse primer "Pax6 exon 56R light" anneals to sequence spanning the junction between exons 5 and 6. The 3' 3 bp of the primer crosses the exon boundary. If exon 5a is present, this primer can not bind. Coloured bars: *Pax6 *exons. Arrows: Q-PCR primers.

**Figure 5:**
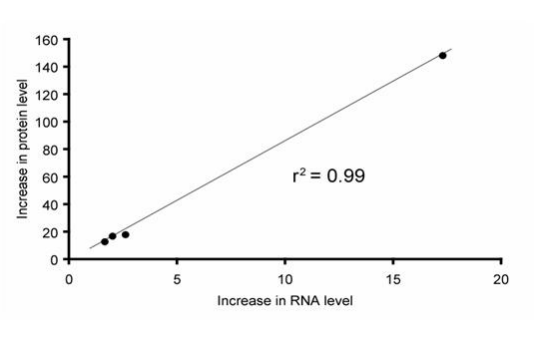
**Correlation between fold increase in mRNA and protein production from the endogenous *Pax6 *locus in Neuro2A cells**. Graph shows data for production of Pax6 following introduction of Pax6(5a) expressing construct and Pax6(5a) following introduction of Pax6 expressing construct.

Quantitative PCR (Q-PCR) with primers specific to either *Pax6 *or *Pax6(5a) *(Fig 4; sequences in Methods) was used to demonstrate upregulation of *Pax6(5a) *mRNA in Neuro2A cells transfected with the Pax6-expressing contruct and *vice-versa*. Figure 5 shows average fold increases in protein levels plotted against average fold increases in mRNA levels for each of the four lines (3 repeats per cell line; increases were relative to the average level in a control line, assigned a value of 1.0). There was a positive correlation between the mRNA increase and the protein increase. These data confirm upregulation of transcription from the endogenous locus in response to introduction of either Pax6 or Pax6(5a) expression constructs.

**Figure 6:**
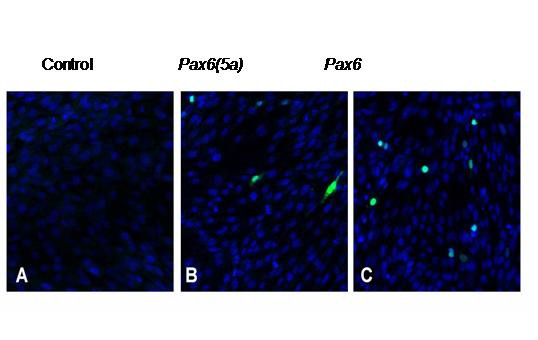
**Transfection of NIH3T3 cells with Pax6(5a) and Pax6 expression constructs**. (A) Untransfected cells do not express Pax6. (B,C) Immunocytochemical detection of Pax6 proteins (green) following transient transfection of with *Pax6(5a) *or *Pax6*.

Pax6 proteins were never detected in untransfected NIH3T3 cells (Fig. 6A) but, as for Neuro2A cells, were found by immunocytochemistry in transfected cells (Fig. 6B,C). Western blots failed to detect Pax6 proteins and RT-PCR failed to detect *Pax6 *mRNA in untransfected NIH3T3 cells. In NIH3T3 cell lines with stable integration of either Pax6 or Pax6(5a) expression constructs, mRNA and protein for both isoforms were detected. Levels varied from line to line but were generally comparable to those in stably transfected Neuro2A lines. Whether the cells contained a Pax6 expressing construct or a Pax6(5a) expressing construct, expression of the Pax6 isoform was always higher than that of the Pax6(5a) isoform. The intensity of the Pax6 and the Pax6(5a) bands in Westerns were measured relative to the highest intensity found, which was assigned a value of 1.0. In cells containing Pax6 expressing constructs, the average intensity of the Pax6 band was 0.64 ± 0.20 (sem) and of the Pax6(5a) band was 0.40 ± 0.15 (n = 6 lines). In cells containing Pax6(5a) expressing constructs, the average intensity of the Pax6 band was 0.51 ± 0.18 and of the Pax6(5a) band was 0.26 ± 0.04 (n = 4 lines). Pax6:Pax6(5a) ratios ranged from 1.4:1 to 4.0:1 in these cell lines. We concluded that the result of expressing either one or other isoform on the endogenous locus was the same in NIH3T3 cells as in Neuro2A cells. Introduction of expression constructs into non-expressing cell lines was sufficient to induce expression from the endogenous locus.

## Discussion

We examined the effect of Pax6 proteins on *Pax6 *expression from its intact endogenous locus, rather than from isolated regulatory sequences, by driving either Pax6 or Pax(5a) protein from an integrated expression construct in cells and measuring the levels of production of both isoforms. We found that in cells containing a construct over-producing either one isoform or the other, production of both forms was increased within the cells. Since increased production of the isoform that was not generated from the introduced construct must have come from the endogenous locus, we concluded that under these conditions PAX6 proteins are capable of positively regulating an intact *Pax6 *locus. In a cell line that does not normally express *Pax6*, introduction of Pax6 or Pax6(5a) was sufficient to activate the endogenous locus. It was interesting to observe in these experiments that the ratio of the two forms remained close to values observed *in vivo *[41-45]. The induction from the endogenous locus of the isoform that was not introduced, which prevents non-physiological distortion of the Pax6:Pax6(5a) ratio, might be revealing the existence of an autoregulatory mechanism controlling the relative levels of the two isoforms.

Pax6 affects several aspects of eye and brain development, including progenitor cell proliferation and neuronal differentiation [9,39] Studies of *Pax6*^-/- ^mice have indicated that Pax6 promotes proliferation during early cortical neurogenesis, but may have the opposite effect in the developing diencephalon, or indeed in the cortex at later stages of neurogenesis [9,17] There is evidence that loss of Pax6 promotes asymmetrical division and production of neurons in the early cortex of *Pax6*^-/- ^mice [9] but that later neurogenesis is impaired in mutants [39]. In the retina, loss of Pax6 accelerates neuronal differentiation [46]. Whereas Pax6 has an early role in patterning the developing nervous system [1,4,6], it later adopts an important function in axon guidance [13,14].

The changing roles of Pax6 with age, and/or differences between its function in different tissues, might be due at least in part to a shift in the relative concentrations of Pax6 and Pax6(5a) [41,47] Even relatively small changes in the ratio might be important since stronger effects on gene activity *via *Pax6 and Pax6(5a) consensus binding sequences are observed if *Pax6 *and *Pax6(5a) *are introduced into cultured cell lines at ratios of 1:1 or 8:1 rather than at ratios of 2:1, 4:1 or 16:1 [47]. There is evidence that the functions of Pax6 and Pax6(5a) differ in vertebrates [25-27], as do their homologues in invertebrates [28,29]. Studies of *Pax6*- and *Pax6(5a)*-related genes in *Drosophila melanogaster*, *ey/toy *and *eyg/toe*, have shown that they promote, respectively, differentiation and proliferation of eye precursor cells [28,29]. Overexpression of Pax6 and Pax6(5a) can alter the expression of different sets of genes in mammals [26,27] Mammalian brain cells reduce their proliferation in response to overexpression of Pax6 or Pax6(5a) and increase their neurogenesis in response to overexpression of Pax6 [25]. Thus, regulation of the relative levels of the two isoforms is very important for normal development.

## Conclusion

Our data show the intact *Pax6 *locus is subject to positive autoregulation and suggest this might provide a mechanism that stabilises the relative levels of the major isoforms of Pax6.

## Methods

### In vitro over-expression studies

Full-length *Pax6 *and *Pax6(5a) *cDNAs, obtained by RT-PCR, were cloned into pCMV-Script (Stratagene) and sequenced in both directions. Neuro2A cells (ECACC 89121404) and NIH3T3 cells (ECACC 93061524) were maintained in Minimal Essential Medium supplemented with 10% foetal bovine serum, 1% non-essential amino acids, 1% L-glutamine and 1% penicillin streptomycin (Gibco). Stable clones were created by transfection (using Lipofectamine 2000; Invitrogen) with linearised *Pax6*, *Pax6(5a) *expression plasmids or a GFP expression plasmid (pEGFPN1; Clontech) followed by selection with G-418 at 500 μg·μl^-1 ^(NIH3T3) and 600 μg·μl^-1 ^(NIH3T3) [37,38] Clones were tested for the anticipated effects of over-expression on neuronal differentiation [39]. Cells were seeded on glass coverslips or directly into polystyrene wells at 2.5 × 10^4 ^cells·cm^-2^. After 72 h, cells were fixed in 4% paraformaldehyde, permeabilised in 100% methanol at -20°C or 0.1% Triton X-100 at room temperature for 10 minutes and reacted with anti-Pax6 (Developmental Studies Hybridoma bank; 1:75) and anti-β-III-tubulin (Sigma; 1:1000) primary antibodies. Secondary antibodies were AlexaFluor 448- and 568- conjugated anti-mouse IgG1 (Molecular Probes; 1:200); cells were counterstained in TOPRO-3 (Molecular Probes; 1:5000) and RNaseA (Sigma; 1:1000).

### Western blots

Total protein was extracted by mechanical homogenisation of confluent cell cultures in 20 mM Tris-HCl, 2 mM EDTA, 150 mM NaCl, 1% Triton-X100 with Complete Protease Inhibitors (Roche) and quantified with the BCA protein assay kit (Pierce). Proteins were resolved by denaturing SDS-PAGE on 12% tris-glycine gels (Invitrogen) and transferred to PVDF membranes (BioRad). Antibodies were anti-Pax6 serum 13 (S. Saule, Institut Curie, Paris, France; 8) and anti β-actin (Sigma; 1:5000) and were detected with the ECL+ chemiluminescent system (Amersham). The densities of bands on X-ray films were quantified using a GS-710 densitometer and Quantity One software (BioRad).

### Quantitative RT-PCR

Quantitative RT-PCRs were performed using an Opticon DNA Engine, to determine the relative levels of *Pax6 *and *Pax6(5a) *mRNA. Q-PCR reactions were optimised to proceed without the formation of primer dimmers or ectopic bands, which would interfere with quantitation. PCRs were carried out in the presence of SYBR Green (Qiagen). After 35 PCR cycles, a melting curve of the PCR product was obtained by measuring the fluorescence emitted at decreasing temperature increments. A smooth sigmoid was an indication that the only double-stranded DNA present in the PCR product was dimerised product, and no primer dimers or ectopic bands were contaminating the reaction. Reaction conditions were optimised until a smooth sigmoidal melting curve was seen after each reaction. A calibration standard was created for each primer set by serial dilution of the most concentrated DNA template. The number of cycles after which the fluorescence of a reaction rose above baseline was designated as the cycle threshold, c(t). As more DNA was included in a reaction, the c(t) dropped, and a calibration curve of volume of DNA against c(t) was plotted. The c(t) of each sample DNA was then plotted onto this calibration curve, thus allowing relative DNA quantitation across PCR reactions.

Primers were designed to allow amplification of the *Pax6 *gene between exons 5 and 6 (Fig. 4). The "Pax6 Exon 5F light" primer (5' GCT TGG TGG TGT CTT TGT CA 3') binds within exon 5 of the *Pax6 *cDNA, and should bind all *Pax6 *cDNA present in a sample. The "Pax6 Exon 56R light" primer (5' TCA CAC AAC CGT TGG ATA CC 3') takes advantage of the fact that primer binding specificity is conferred by 3' sequence, to allow selective amplification of the *Pax6 *splice form. 19 bp of this primer corresponds to sequence in the 5' end of exon 6. The most 3' residue, however, binds to the G residue at the 3' end of exon 5. Thus, this primer can only bind target cDNA when exon 5 is spliced directly onto exon 6, and exon 5a is absent. The "Pax6 Exon 55aR light" primer (5' TTT GCA TCT GCA TGG GTC T 3') spans the junction between exons 5 and 5a, and can only bind to the *Pax6(5a) *splice form. Product size were 131 bp for the Exon 5F light/Exon 56R light pair and 129 bp for the Exon 5F light/Exon 55aR light pair.

## Abbreviations

GFP: Green fluorescent protein

Q-PCT: Quantitative polymerase chain reaction

## Authors' contributions

J. Pinson carried out the experiments and wrote a draft of the paper. T.I. Simpson helped design and carry out the experiments. J.O. Mason and D.J. Price helped design and supervise the work and write the paper.
